# Transcriptional profile and chromatin accessibility in zebrafish melanocytes and melanoma tumors

**DOI:** 10.1093/g3journal/jkab379

**Published:** 2021-11-15

**Authors:** Eva T Kramer, Paula M Godoy, Charles K Kaufman

**Affiliations:** Division of Medical Oncology, Departments of Medicine and Developmental Biology, Washington University in Saint Louis, St Louis, MO 63110, USA

**Keywords:** zebrafish, melanocytes, melanoma, RNA-seq, ATAC-seq

## Abstract

Transcriptional and epigenetic characterization of melanocytes and melanoma cells isolated from their *in vivo* context promises to unveil key differences between these developmentally related normal and cancer cell populations. We therefore engineered an enhanced *Danio rerio* (zebrafish) melanoma model with fluorescently labeled melanocytes to allow for isolation of normal (wild type) and premalignant (*BRAF^V600E^*-mutant) populations for comparison to fully transformed *BRAF^V600E^*-mutant, *p53* loss-of-function melanoma cells. Using fluorescence-activated cell sorting to isolate these populations, we performed high-quality RNA- and ATAC-seq on sorted zebrafish melanocytes *vs.* melanoma cells, which we provide as a resource here. Melanocytes had consistent transcriptional and accessibility profiles, as did melanoma cells. Comparing melanocytes and melanoma, we note 4128 differentially expressed genes and 56,936 differentially accessible regions with overall gene expression profiles analogous to human melanocytes and the pigmentation melanoma subtype. Combining the RNA- and ATAC-seq data surprisingly revealed that increased chromatin accessibility did not always correspond with increased gene expression, suggesting that though there is widespread dysregulation in chromatin accessibility in melanoma, there is a potentially more refined gene expression program driving cancerous melanoma. These data serve as a resource to identify candidate regulators of the normal *vs.* diseased states in a genetically controlled *in vivo* context.

## Introduction

Human melanoma is notable as one of the most highly mutagenized cancers, making comparisons between patient samples difficult given variation in potentially irrelevant passenger somatic mutations in addition to the background of an outbred heterogenous population (Hodis *et al.* 2012). Genetically engineered tumor models allow for comparisons of premalignant and cancer cells between highly related individuals (*e.g.*, siblings, cousins) with defined driver and tumor suppressor mutations. A zebrafish model combining a BRAF^V600E^ mutation, present in over 50% of human melanomas, with a loss of function (lf) in *p53* develops one to three melanomas in its lifetime (Patton *et al.* 2005). Despite high genetic relatedness, the Tg(*BRAF^V600E^)/p53^lf/lf^* tumors still display genomic heterogeneity but without clear functional consequences in this model (Yen *et al.* 2013). High heterogeneity in melanoma can lead to variable responses to currently available therapies (Reuben *et al.* 2017). To continue to identify novel melanoma therapies, we must delve deeper into the transcriptional and epigenetic differences that exist between the normal and diseased states.

Many studies have utilized next-generation sequencing technologies to evaluate melanoma subtypes based on clinicopathological characteristics (Cancer Genome Atlas Network 2015; Hayward *et al.* 2017; Tsoi *et al.* 2018; Rabbie *et al.* 2019; Cisarova *et al.* 2020; Durante *et al.* 2020), major mutations (Cancer Genome Atlas Network 2015; Travnickova *et al.* 2019), and drug resistance and survival (Rambow *et al.* 2018; Garg *et al.* 2021). Prior studies comparing melanocytes and cutaneous melanoma cells have reported on coding mutations, gene expression changes only, or have used human or zebrafish cell lines rather than focusing on *in vivo* animal models (Yen *et al.* 2013; Haltaufderhyde and Oancea 2014; Kaufman *et al.* 2016; Badal *et al.* 2017; Kunz *et al.* 2018; Venkatesan *et al.* 2018; Marie *et al.* 2020; Wouters *et al.* 2020). Single-cell RNA-seq experiments so far generally focus on the heterogeneity within cell populations, rather than a comparison between melanocytes and melanoma cells, or they do not evaluate the epigenetic landscape (Ennen *et al.* 2015; Tirosh *et al.* 2016; Gan *et al.* 2018; Rambow *et al.* 2018; Baron *et al.* 2020; Li *et al.* 2020; Belote *et al.* 2021; Travnickova and Patton 2021). Thus, we sought to characterize the epigenetic and transcriptional differences between zebrafish nonmalignant, precancerous, and malignant melanocytes in a genetically defined melanoma context.

Since its initial report in 2013, Assay for Transposase Accessible Chromatin with high-throughput sequencing (ATAC-seq) has become a widely used method to provide a sensitive assessment of genomic accessibility (Buenrostro *et al.* 2013). The combination of epigenetic and transcriptional approaches has been used to decipher regulators in hematopoiesis and leukemia (Corces *et al.* 2016), find loci influencing pancreatic α- and β-cell differentiation (Ackermann *et al.* 2016), identify regions influencing gestational duration (Sakabe *et al.* 2020), characterize intermediate states in melanoma cell cultures (Wouters *et al.* 2020), among many other applications. Still, a comparison of the epigenetic and transcriptional profiles of sorted normal melanocytes to melanoma tumors in a native, *in vivo* biological context utilizing an animal model has been limited, thus diminishing our ability to dissect mechanisms governing melanoma initiation.

Animal models represent a powerful tool to understand disease initiation and progression, discover therapeutic targets, and test drugs. Utilizing the Tg(*BRAF^V600E^)/p53^lf/lf^* zebrafish model, we probed the natural biological context of normal melanocytes and related disease development, specifically that of melanoma cancer, using RNA- and ATAC-seq (Patton *et al.* 2005; White *et al.* 2011; Kaufman *et al.* 2016). Furthermore, we compared the transcriptional profiles of zebrafish melanocytes (MC) and melanoma cells (MA) to existing human RNA-seq datasets classifying subtypes of melanoma (Jönsson *et al.* 2010; Harbst *et al.* 2012; Cancer Genome Atlas Network 2015; Cirenajwis *et al.* 2015; Nsengimana *et al.* 2015; Lauss *et al.* 2016; Badal *et al.* 2017; Gan *et al.* 2018; Kunz *et al.* 2018) to further confirm the relevance of these animal models. We present a transcriptomic and genome-wide chromatin accessibility analysis of precancerous MC and fully transformed MA in the most widely used zebrafish melanoma model to be used as a resource for identifying pathways, genes, and loci which differentiate melanoma from normal pigment cells.

## Materials and methods

### Zebrafish husbandry

Zebrafish were raised in the Washington University Zebrafish Consortium in accordance with animal protocols and the Washington University IACUC. Pair or harem crosses generated embryos, which were then raised at 28.5°C. The following wild type (WT), mutant, and transgenic strains were utilized: AB*, *Tg(MiniCoopR; mitfa:mCherry)*, *Tg(mitfa:BRAF^V600E^)*; *p53*^lf/lf^*; Tg(crestin:EGFP)*, *Tg(mitfa:BRAF^V600E/+^); p53*^+/lf^*; mitfa*^+/−^*; Tg(MiniCoopR; mitfa:mCherry)*, *Tg(mitfa:BRAF^V600E^)/p53^lf/lf^/mitfa^+/^*^*−*^*/Tg(MiniCoopR; mitfa:mCherry)* and *Tg(mitfa:BRAF^V600E^); p53*^lf/lf^*; mitfa*^−/−^.

### Transgenic fish generation

First, we injected WT AB* zebrafish with *MiniCoopR:mitfa:mCherry*. Fish with germline transmission of *Tg(MiniCoopR; mitfa*:*mCherry)* (source of MC_WT) were then crossed to *Tg(mitfa:BRAF^V600E^); p53*^lf/lf^*; mitfa*^−/−^ zebrafish and scored for presence of mCh*+* melanocytes. These *Tg(mitfa:BRAF^V600E/+^); p53*^+/lf^*; mitfa*^+/−^*; mitfa:mCherry* fish (source of MC_Het) were crossed to *Tg(mitfa:BRAF^V600E^); p53*^lf/lf^*; mitfa*^−/−^ zebrafish and scored for presence of mCh*+* melanocytes, and PCR genotyped for BRAF and p53 status to confirm generation of *Tg(mitfa:BRAF^V600E^)/p53^lf/lf^/mitfa^+/−^/mitfa:mCherry* zebrafish (source of MC_Homo).

### Tissue collection

For RNA-seq, we utilized mCh+ melanocytes sorted from the skin of three independent AB* zebrafish expressing *MiniCoopR:mitfa:mCherry*, three independent zebrafish heterozygous for Tg(*BRAF^V600E/+^)/p53^+/lf^/mitfa^+/−^*, and GFP+ melanoma tumor cells from five distinct tumors from three *Tg(BRAF^V600E^)/p53^lf/lf^/Tg(crestin:EGFP)* fish. The melanoma samples from MA3A, MA3B, and MA3C were isolated from one fish with three tumors: a tumor near the dorsal fin, a tumor on the tail, and a tumor on the head. For ATAC-seq, we utilized mCh+ melanocytes from three zebrafish heterozygous for Tg(*BRAF^V600E/+^)/p53^+/lf^/mitfa^+/−^* and one zebrafish with homozygous BRAF/p53 status [genotype Tg(*BRAF^V600E^)/p53^lf^/mitfa^+/−^*], and GFP+ melanoma tumor cells from eight *Tg(BRAF^V600E^)/p53^lf/lf^/Tg(crestin:EGFP)* fish.

Animals were euthanized using approved methods and bulk nodular tumors were excised using a razor blade. Skin samples were isolated by decapitating the euthanized zebrafish and peeling the skin off the muscle using two sets of forceps. Tumor or skin samples were manually sheared with a shortened pipette tip or using a homogenizer and then incubated in fresh 0.9× PBS with 12.5 µg/ml liberase for up to 30 min to dissociate cells. Fetal bovine serum terminated the reaction and the cells were passed through a 40 mm filter. Cells were centrifuged at 2000 × g for 5 min at 4°C. Supernatant was removed and the cells were resuspended in 500 µl 0.9× PBS and kept on ice. Fluorescence-activated cell sorting (FACS) was performed by the Washington University in St Louis core facility to isolate GFP+ tumor cells from sorted tumor samples or mCh+ melanocyte cells from sorted skin samples. Unpigmented and pigmented GFP+ and GFP− tumors were utilized to set the gating for GFP+ melanoma cells. Skin from mCh− AB* zebrafish was used to set the gating for isolating mCh+ melanocytes. Briefly, we gated first to exclude debris and doublets, then gated for the desired fluorescent marker based on comparison to a nonfluorescent sample.

### Imaging

Images were acquired using a Nikon SMZ-18 with RiD2 color camera. For whole adult zebrafish images, multiple images were taken at 0.75× magnification and then merged in Adobe Lightroom 2020. Magnification of additional images for Supplementary Figure 1 specified in figure legend.

### RNA library preparation and sequencing

RNA was isolated from cells using the Machery Nagel Nucleospin XS kit (Fischer Scientific cat No. NC0389511). Quantity and quality of RNA were assessed using a High Sensitivity RNA ScreenTape on an Agilent 2200 TapeStation. Sample preparation for sequencing was performed by the Genome Technology Access Center (GTAC). Briefly, cDNA was generated using a Clontech SMARTer cDNA amplification kit as per manufacturer’s recommendation. 1× 50 base pair single-end sequencing was performed on an Illumina HiSeq 3000.

### RNA-seq analysis

Demultiplexed fastq files were provided by the GTAC. Gene set enrichment analysis (GSEA) was performed using the GSEA software (Mootha *et al.* 2003; Subramanian *et al.* 2005). Quality of sequenced reads was assessed by FastQC (Andrews 2010). Reads were aligned to GRCz11/danRer11 by STAR with the following parameters: –outSAMtype BAM SortedByCoordinate –outFilterType BySJout –outSAMunmapped Within –outSAMattrIHstart 0 –outFilterIntronMotifs RemoveNoncanonical –quantMode TranscriptomeSAM –outSAMstrandField intronMotif (Dobin *et al.* 2013). Transcriptome quantification was performed by RSEM using the danRer11 NCBI RefSeq gene annotations (Li and Dewey 2011). RNA-seq reads were normalized with DESeq2 (Anders and Huber 2010). Boxplots were made using ggplot2 (Wickham 2016), heatmaps with either ggplot2 or the pheatmap function (Kolde 2015), and principal component analysis (PCA) was performed using the plotPCA function in the DESeq2 package (Anders and Huber 2010).

### ATAC-seq library preparation and sequencing

Up to 50,000 cells per sample were tagmented with Nextera Tn5 transposase using the Illumina Nextera kit and purified with a Qiagen MinElute reaction kit per methods modified from Buenrostro *et al.* (2013). The DNA was then PCR amplified to add indexing primers in nine cycles. SPRI AMPure beads enriched for fragments under ∼600 bp. The library was then amplified again with the nine-cycle protocol, followed by cleanup with SPRI AMPure beads. The DNA library was quantified with a Qubit DNA High Sensitivity assay and analyzed for quality and size distribution on an Agilent 2200 TapeStation with a High Sensitivity D5000 ScreenTape. Samples were pooled at a 10 nM final concentration. Sequencing was run with an Illumina HiSeq 2500 system with 2 × 50 bp read length by the Washington University in St Louis School of Medicine GTAC.

### ATAC-seq analysis

Demultiplexed fastq files were provided by GTAC. Quality of fastq files was assessed by FASTQC (Andrews 2010). Adapter sequences were removed using CutAdapt (Martin 2011), and reads were aligned to GRCz11/danRer11 using Burrows–Wheeler Aligner using default parameters (Li and Durbin 2009). BAM files were sorted using samtools, and reads with Mapping Quality (MAPQ) scores <30 and improperly paired were removed using bamtools filters with the following commads: -mapQuality “>30” -isProperPair “true” (Li *et al.* 2009; Barnett *et al.* 2011). We used the MarkDuplicates command from the Genome Analysis Tool Kit to remove duplicates (McKenna *et al.* 2010). BAM files were converted to the BED format using bedtools bamtobed command (Quinlan and Hall 2010) and used as input for peak calling with MACS2 (Zhang *et al.* 2008). We used the callpeak command with the following parameters: –g 1e9, –nomodel, –extside 100, –shift –50, –keep-dup 999, –call-summits. Output bedGraph files were sorted, clipped, and converted to BigWig format using the bedGraphToBigWig command (Kent *et al.* 2010). Quality of peaks was assessed by ChIPQC (Carroll *et al.* 2014). The irreproducible discovery rate (IDR) was performed between replicates using the idr command (Li *et al.* 2011). Differentially accessible regions were called using DiffBind (Stark and Brown 2011). All motif analyses were performed using Homer (Heinz *et al.* 2010).

### Histology

Three *Tg(mitfa:BRAF^V600E^)/p53^lf/lf^/mitfa^+^*^*−*^*/mitfa:mCherry* zebrafish and three *Tg(mitfa:BRAF^V600E^);* *p53*^lf/lf^/*crestin:EGFP)* zebrafish were euthanized and each whole fish was placed in a 50 ml conical with 40 ml 10% formalin for fixation and shipped to HistoWiz for further preparation. Briefly, zebrafish were decalcified and embedded; then, five sagittal sections along the midline were stained with hematoxylin and eosin.

## Results

### Isolating melanocyte and melanoma populations from adult zebrafish skin

**Figure 1:**
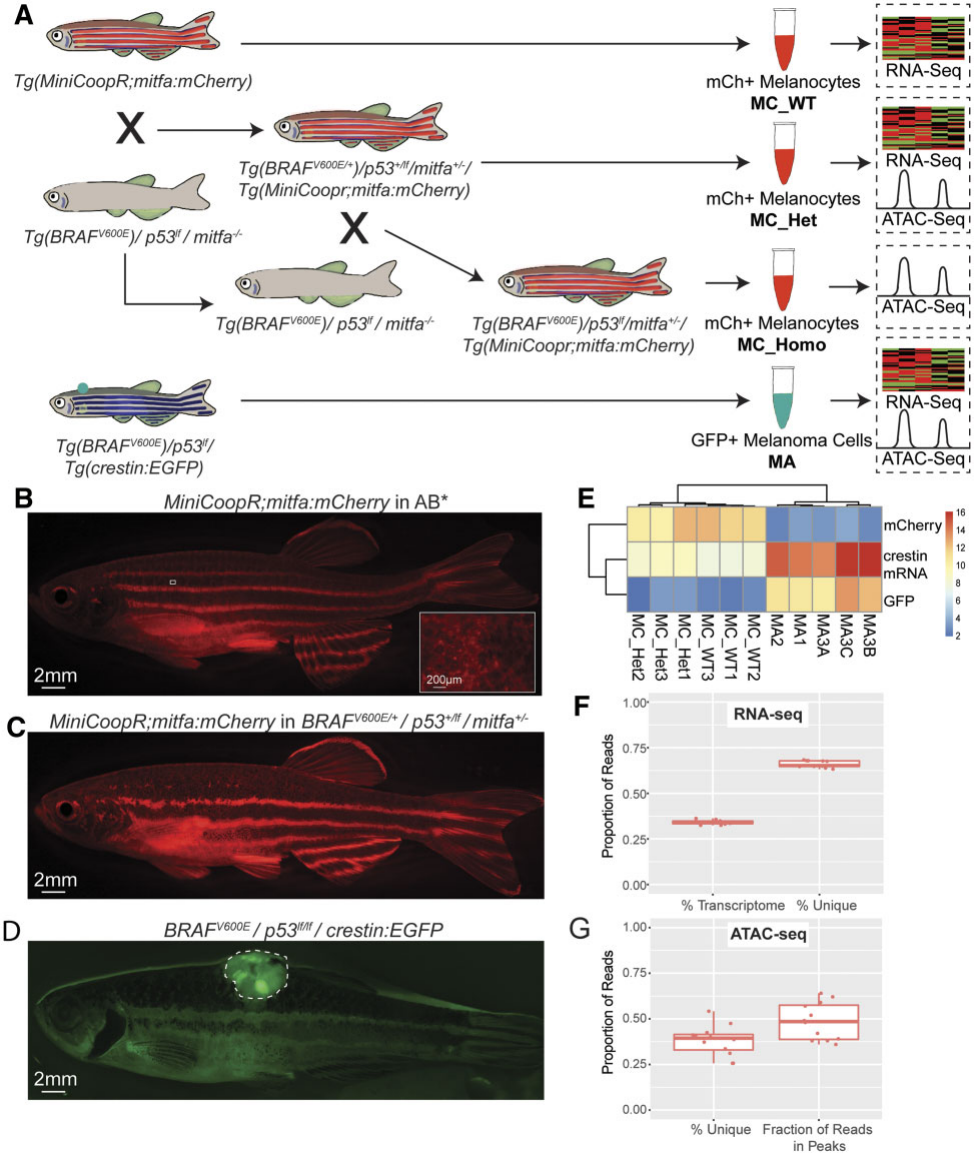
Isolation of zebrafish melanocytes and melanoma cells for RNA- and ATAC-seq analysis. (A) Schematic depicting experimental set up with relevant genotypes, crosses, and sources for each analysis. (B, C) Representative image of zebrafish with *mCherry* labeled melanocytes (note 7× high magnification inset with melanocytes with central dark pigment and visible surrounding fluorescence) and *EGFP* labeled melanoma tumor (D, outlined). (E) Heat map quantifying *mCherry*, *crestin*, and *EGFP* log_2_ read counts in each RNA-seq sample. (F) Quantification of proportion of unique and transcriptomic reads for RNA-seq. (G) Quantification of unique reads in ATAC-seq, and the fraction of reads located in peaks. MC, melanocyte sample; Het, heterozygous for *BRAF^V600E^* and *p53* transgenes; WT, wild-type AB* fish; MA, melanoma sample.

To compare the transcriptional and chromatin accessibility states of normal (WT) and precancerous melanocytes (MC; harboring the *BRAF^V600E^* mutation and *p53* lf mutation) to fully transformed melanoma (MA) cells, we generated a stable transgenic zebrafish line with fluorescently labeled melanocytes expressing mCherry driven by the melanocyte-specific *melanocyte inducing transcription factor a* (*mitfa)* promoter (Ceol *et al.* 2011; White *et al.* 2011; Kaufman *et al.* 2016). A stable *MiniCoopr; mitfa:mCherry* transgenic line was generated in WT AB* zebrafish using *Tol2*-mediated insertion (Figure 1, A and B) and then crossed to *Tg*(*BRAF^V600E^*)*/p53^lf^/mitfa*^*−/−*^ zebrafish (Ceol *et al.* 2011) to generate *Tg*(*BRAF^V600E/+^*)*/p53^+/lf^/mitfa^+/−^/Tg(MiniCoopr; mitfa:mCherry)* zebrafish to investigate the impact of overactivation of *BRAF* in melanocytes (Figure 1, A and C and Supplementary Figure S1). Zebrafish heterozygous at the key *BRAF* and *p53* melanoma driver loci had thicker and darker melanocyte stripes, with minor melanocyte expansion into the interstripe as compared with WT AB* fish (Figure 1, B and C and Supplementary Figure S1), consistent with the melanocyte pattern seen with homozygous *BRAF^V600E^* shown previously (Patton *et al.* 2005). The presence of the *mitfa* minigene in the MiniCoopR backbone containing the *mitfa:mCherry* transgene did not appear to alter the normal striping pattern (whereas the presence of the BRAF and p53 alterations did, comparing Figure 1, B and C), and we detected upregulation of *mitfa* in the MA *vs.* both MC populations (Supplementary Table S1), consistent with prior expression studies (Kaufman *et al.* 2016).

Using FACS, we carefully isolated mCherry-positive (mCh+) melanocytes from *Tg*(*MiniCoopr; mitfa:mCherry)* (MC_WT) and *Tg*(*BRAF^V600E/+^)/p53^+/lf^/mitfa^+/−^/Tg(MiniCoopr; mitfa:mCherry)* (MC_Het) zebrafish skin (Supplementary Figure S2, A, D, and E). We could visualize mCh+ melanocytes in both isolated scales and in the underlying hypodermis when overlying scales were removed (Supplementary Figure S2, C and D), and as we stripped the skin in its entirety after euthanasia, our melanocyte population likely includes both scale associated and hypodermal sources. mCh+ melanocyte cells were similarly ∼1–5% abundant during FACS from the skin of AB* or *BRAF^V600E/+^/p53^+/lf^/mitfa^+/^*^*−*^ zebrafish (Supplementary Figure S2). We also performed FACS on previously described Enhanced Green Fluorescent Protein (EGFP)-positive (GFP+) melanoma tumors from *Tg(BRAF^V600E^)/p53^lf/lf^/crestin:EGFP* (MA) zebrafish to isolate melanoma cells (Figure 1D and Supplementary Figure S2, B, C, F, and H; Kaufman *et al.* 2016). As previously described, these melanoma tumors are identifiable as raised, highly cellular masses that are locally invasive (Supplementary Figure S2, G and H) and readily differentiated from normal skin regions, both grossly (Figure 1, B–D) and on histological sections (Supplementary Figure S2, G and H). Zebrafish *crestin* is expressed in embryonic neural crest (NC) and is re-expressed with emergence of melanoma (Kaufman *et al.* 2016). We then performed RNA- and ATAC-seq with each cell population (see *Materials and* *methods*; Figure 1A and Supplementary Table S2).

As expected, GFP+ melanoma tumor cells and mCh+ melanocyte samples clustered based on *crestin, EGFP*, and *mCherry* normalized read counts by Euclidean distance. *GFP+* samples show high *crestin* expression supporting the fidelity of the *crestin*:*EGFP* transgene as a faithful reporter of endogenous *crestin* expression (Figure 1E). Although melanocytes had some reads aligning to *crestin*, *crestin* expression was 109 times higher in melanoma (39,232 average reads) than in melanocytes (359 average reads), consistent with previously reported massive upregulation of *crestin* in melanoma tumors (White *et al.* 2011; Kaufman *et al.* 2016; Figure 1E).

ATAC-seq and RNA-seq libraries from sorted cells passed established Encode QC metrics for library quality and read depth. Samples for RNA-seq were sequenced to an average of 29,103,353 reads, of which 9,897,358 (34%) aligned to the transcriptome (Figure 1F). Of all genes with at least 1 count per million (CPM) mapped reads, the majority (∼55%) had >10 CPM, suggesting adequate read depth. Similarly, ATAC-seq samples were sequenced to an average of 35,69,8542 paired reads of which 13,594,823 (38.1%) uniquely mapped to the genome (Figure 1G). The average fraction of uniquely mapped reads in peaks is 39.2% (Figure 1G).

### Transcriptomic comparison of normal melanocytes, BRAF/p53 mutant melanocytes, and melanoma cells

**Figure 2:**
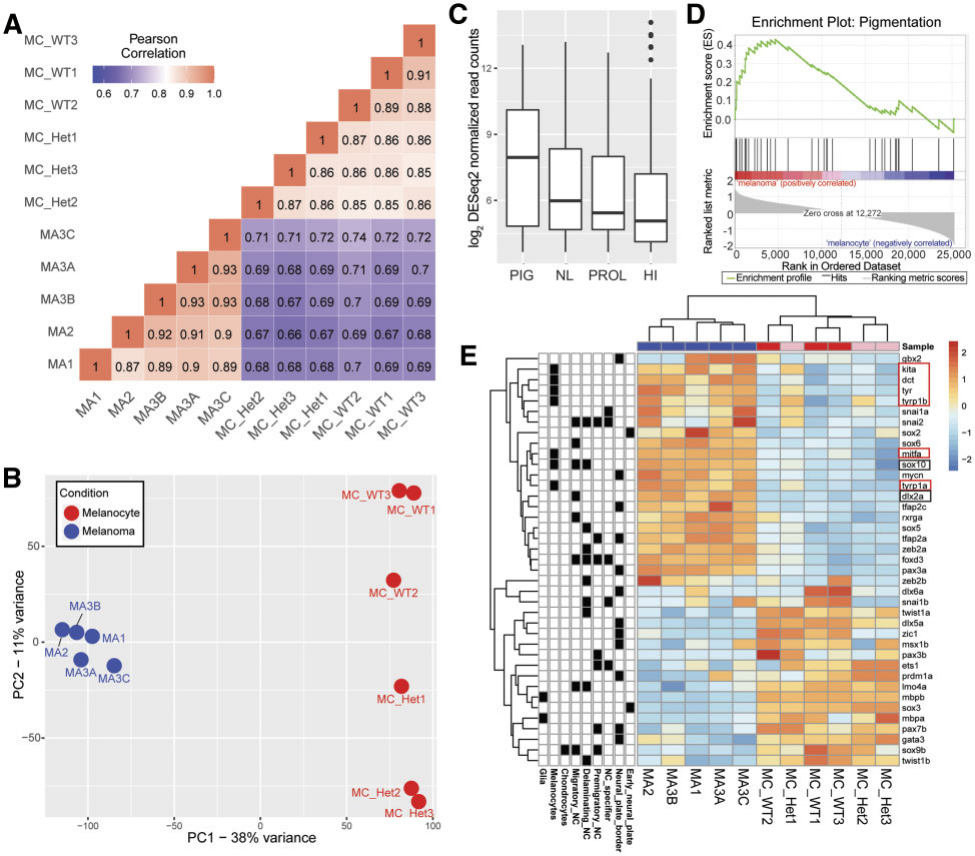
RNA-seq analysis of melanocyte and melanoma cell populations. (A) Correlation plot depicting the Pearson correlation between each sample. (B) PCA plot with the components with the highest variation. (C) Box plot comparing zebrafish ortholog expression in melanoma cells of genes associated with 4 subtypes of human melanoma (Jönsson *et al.* 2010). PIG, pigmentation (median = 7.950548); NL, normal-like (median = 5.982954); PROL, proliferative (median = 5.43669); HI, high immune (median = 5.063983). (D) Positive correlation using GSEA of genes associated with the pigmentation subtype of human melanoma and zebrafish upregulated melanoma genes. Enrichment score = 0.4299, *P*-value = 0.036, *q*-value = 0.134. (E) Heat map depicting relative gene expression between zebrafish melanoma and melanocyte populations for NC genes associated with specific NC populations (*e.g.*, early NC, neural plate border) or descendant lineages (*e.g.*, glia, chondrocytes, melanocytes). NC genes of note in black boxes. Select melanocyte genes in red boxes. MC, melanocyte sample; Het, heterozygous for *BRAF^V600E^* and *p53* transgenes; WT, wild-type AB* fish; MA, melanoma sample.

Transcriptional profiles of GFP+ (melanoma) were highly correlated (Pearson *R* ≥ 0.85; Figure 2A), even from different fish. mCh+ (melanocytes) samples also had highly correlated transcriptional profiles (Figure 2A). Melanocytes from WT fish (MC_WT) and fish heterozygous for the BRAF^*V600E*^-driver oncogene and *p53* lf allele (MC_Het) were also highly correlated with one another with small differences in correlation coefficients between origin genotype, which is notable given the presence of the activated *BRAF^V600E^* oncogene in the MC_Het melanocytes (*R* = 0.88–0.91 within WT fish, *R* = 0.85–0.87 within heterozygotes, and *R* = 0.85–0.87 across genotypes; Figure 2A). Melanoma samples were similarly highly correlated (*R* = 0.87–0.93) with slightly higher correlation for multiple tumors isolated from the same fish (*R* = 0.93 for MA3A, MA3B, MA3C, and *R* = 0.87–0.92 for external replicates). Melanocytes and melanoma cells readily separated using unsupervised hierarchical clustering (correlation coefficients between 0.74 and 0.81) indicating that transcriptional profiles alter during transformation from a *BRAF* mutant melanocyte to a fully oncogenic melanoma cell, but remain more closely correlated in a WT (MC_WT) or premalignant (MC_Het) state. Furthermore, PCA readily separates melanocytes from melanomas, with the first principal component accounting for 38% of the variance (Figure 2B). Principal component two (accounting for 11% of the variance) likely captures the lesser transcriptional differences between the WT and Het MC samples.

We identified 1144 genes significantly upregulated in melanomas *vs.* melanocytes, and 2984 genes significantly upregulated in melanocytes compared with melanoma cells [log_2_ fold change (log_2_FC) > 1 and adjusted *P*-value < 10^−6^, Supplementary Figure S3A]. Surprisingly, the number of melanoma-upregulated genes meeting increasing log fold change cutoffs dropped off more steeply than melanocyte-upregulated genes, indicating that more genes are downregulated in melanoma as compared with melanocytes (Supplementary Figure S3B).

Comparing WT and Het (*Tg(BRAF^V600E/+^)/p53^+/−^*) melanocytes, there are far fewer significantly differentially expressed genes with 12 genes upregulated in WT over Het melanocytes and 41 genes upregulated in Het over WT melanocytes with log_2_FC > 1 and adjusted *P*-value ≤ 10^−6^ (Supplementary Figure S3C). These results highlight the surprising similarity within melanocytes and premalignant (*i.e.*, BRAF mutant/p53 mutant) melanocytes as compared with the more dramatic changes that occur when compared with transformed melanocytes/melanoma. To facilitate further inquiry, we provide a list of all gene expression changes and *P*-values between melanocytes, premalignant melanocytes, and melanoma cells (Supplementary Table S1).

### Gene expression programs

We compared gene expression profiles between our melanocyte and melanoma samples to the four gene lists from Jönsson *et al.* (2010) stratifying human cutaneous melanoma into proliferative, pigmentation, high immune, and normal-like subtypes. First, we assessed which subtype of human melanoma our zebrafish melanoma samples most resembled and found the highest average expression of genes associated with the pigmentation subtype (Figure 2C). This was further confirmed with GSEA detecting significant enrichment of genes associated with the human melanoma pigmentation subtype (GSEA ES = 0.4299, *P* = 0.036; Figure 2D). Finally, comparing expression in zebrafish melanocytes to melanoma cells, genes in the pigmentation list had the most significant upregulation in melanoma cells (paired *P*-value = 0.007; Supplementary Figure S3, D–H).

Previous studies have shown that melanoma reactivates aspects of an embryonic NC program, with prominent NC genes such as *sox10* the subject of much interest (Shakhova *et al.* 2012; Cronin *et al.* 2013; Mohamed *et al.* 2013; Kaufman *et al.* 2016). The central gene regulatory network, or “wiring diagram,” for NC development has been well-described, and we used the NC gene regulatory network described by Kunz *et al.* (2018) to examine gene expression changes in each condition for NC pathway genes (Supplementary Table S3). We found *sox10* and *dlx2a* are upregulated in melanoma in agreement with previous smaller scale transcriptional analyses (black boxes, Figure 2E; Kaufman *et al.* 2016). Interestingly, important melanocyte-specific genes including *mitfa, tyrp1b, kita, dct, tyr*, and *tyrp1a* were upregulated in the melanoma samples, supporting that melanoma cells sustain a continued presence of an active melanocyte identity (red boxes, Figure 2E). To further probe this finding, we assessed whether either cell population had enrichment of human melanocyte genes (Reemann *et al.* 2014). Indeed, both zebrafish melanocytes and melanoma cells had high expression of human melanocyte genes (Supplementary Figure S3H).

Another recurring and important question relates to the idea that the NC incorporates a spectrum of developmental time (*e.g.*, from specification, to delamination, to migration, etc.), spatial differences (*e.g.*, cranial, thoracic, caudal NC), and descendant lineages (*e.g.*, glia, melanocyte, chondrocyte), each with defining genetic markers in its transcriptional program (Supplementary Table S3; Simões-Costa and Bronner 2015; Williams *et al.* 2019). We reviewed our melanoma differential gene sets in relation to these NC subsets and, aside from a “strengthening” of the melanocyte program as noted above and decrease in expression of glial (*mbpa/b*) and chondrocyte (*sox9b*) factors, we did not note other clear unifying NC subsets that aligned more closely to the melanoma program (Figure 2E). Given the widespread dysregulation of gene expression in the cancerous melanoma state, this is perhaps unsurprising that the malignant phenotype would entail a broad and not entirely faithful amalgamation of melanocyte and NC transcriptional identities.

### Chromatin accessibility profiles in melanocytes and melanoma cells

**Figure 3:**
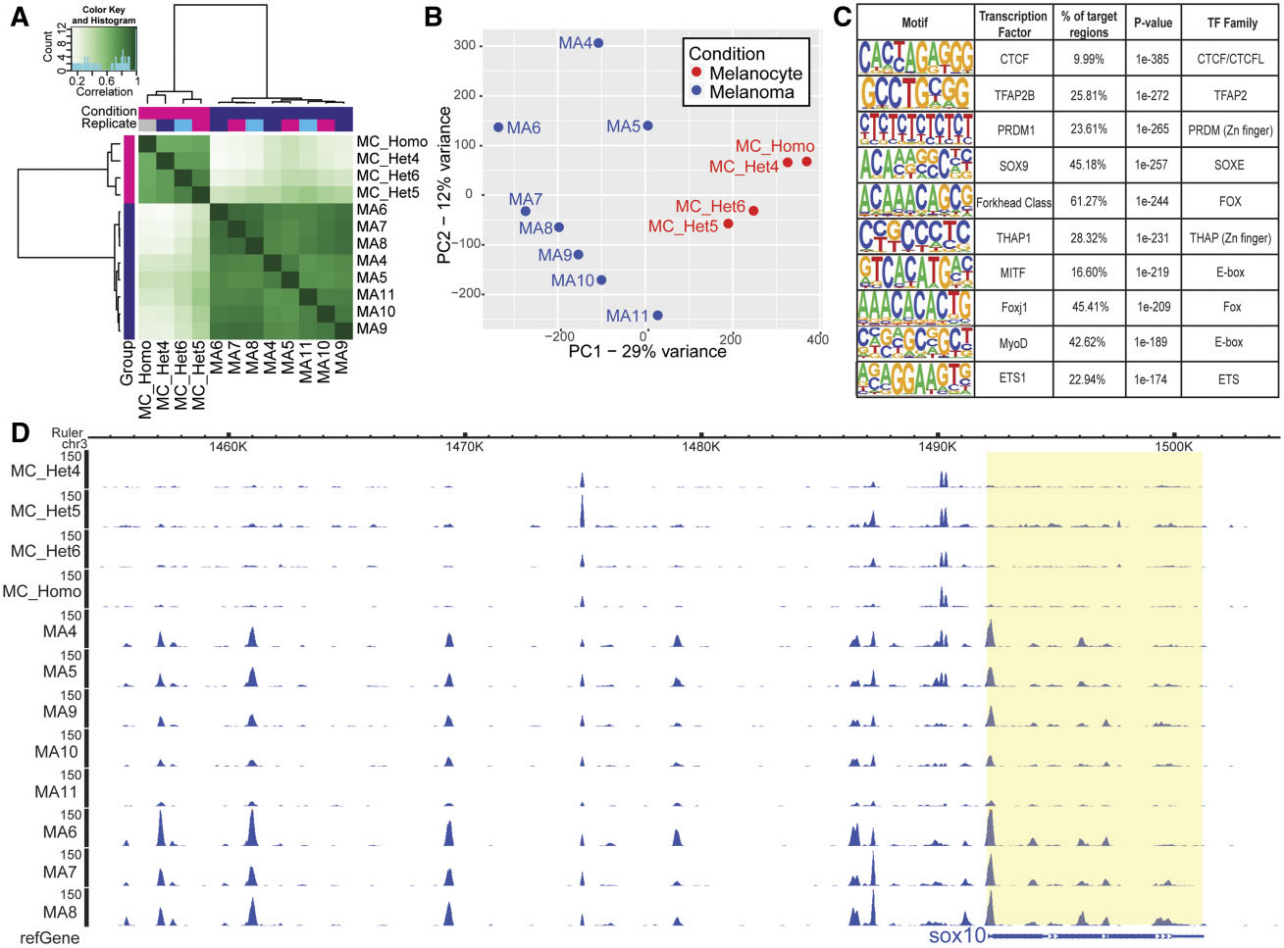
ATAC-seq analysis of melanocyte and melanoma cells. (A) Correlation plot of overall chromatin accessibility across the genome, with darker green color indicating greater correlation between samples. (B) PCA plot comparing similarity of chromatin accessibility across conditions. (C) Predicted TF binding sites, based on HOMER analysis, enriched in open chromatin domains more accessible in melanoma *vs.* melanoma cells. Each TF is reported with its associated TF family, as TF families frequently have similar or identical binding sites which are not differentiated by HOMER. (D) Epigenome browser tracks near and upstream of the NC and melanoma gene *sox10* on chromosome 3. MC, melanocyte sample; Het, heterozygous for *BRAF^V600E^* and *p53* transgenes; Homo, homozygous for *BRAF^V600E^* and *p53* transgenes; MA, melanoma sample.

To characterize the genome-wide chromatin accessibility of premalignant *Tg(BRAF^V600E^)/p53^lf/lf^* mutant melanocytes and melanoma, we isolated premalignant melanocytes (mCh*+*) from four zebrafish, including three MC_Het and one sample from a *Tg(BRAF^V600E^)/p53^lf/lf^/Tg(MiniCoopR; mitfa:mCherry)* zebrafish (MC_Homo). Since MC_WT and MC_Het read counts were highly correlated in the RNA-seq, we utilized MC_Het zebrafish for ATAC-seq analysis. We compared these profiles to those from eight melanoma tumors sorted for *crestin:EGFP* expression (Figure 3A).

As in the RNA-seq analysis, melanocyte samples and melanoma samples clustered according to normal *vs.* malignant state (Figure 3, A and B). To assess the reproducibility of each identified accessible site within a condition, we evaluated the IDR, setting a threshold of 0.05. For MC samples, an average of 53% of sites passed IDR 0.05, and for the MA samples, the average was 33% (Supplementary Figure S4A).

In our ATAC-Seq analysis, 56,936 sites demonstrated differential accessibility (*P* < 0.05, IDR < 0.05) between melanocyte and melanoma cells. Open regions, or peaks, within 3 kilobases (kb) of transcriptional start sites (TSS) were centered at the TSS (Supplementary Figure S4B). Of promoter annotated peaks, 85% of differentially accessible sites were more open in melanoma as compared with melanocytes (Supplementary Figure S4C and Table S4).

### Open chromatin regions and putative transcription factor binding sites

We examined the differentially accessible chromatin regions using HOMER *de novo* analysis to identify over-represented DNA motifs representing putative transcription factor (TF) binding sites (Figure 3D). In order of significance, sites associated with CCCTC-binding factor (CTCF) topped the list—a TF commonly mutated in cancer and known to normally function widely in controlling chromatin architecture (Kim *et al.* 2015). TF binding motif families corresponding to key NC-associated factors also rounded out the top 10 list, including the Forkhead, SOXE, and ETS families. Melanocyte and NC programs are particularly active in melanoma cells, with increased *sox10* and *mitfa* expression, respectively, seen in the RNA-seq data (Figure 2E), and this appears consistent with enrichment of SOX9/10 (SOXE family) and MITF (E/M-boxes) binding sites in more accessible regions in melanoma (Figure 3C). Finally, we noted the overall similarity in the contour of chromatin accessibility within premalignant melanocytes and melanomas as well as the numerous areas of differential accessibility as, for example, near *sox10* and *mitfa* (Figure 3D and Supplementary Figure S4D).

### Modeling gene expression as a function of peak accessibility

**Figure 4.**
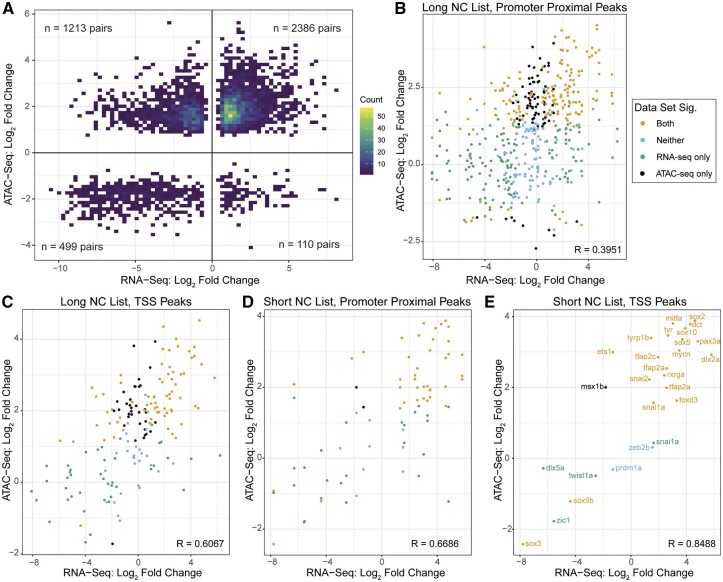
Integration of gene expression and accessibility. (A) Significantly differentially expressed genes between melanoma and melanocyte cells (RNA-seq, |log_2_FC| > 1, *P* < 0.05) plotted against the most differentially accessible peak (ATAC-Seq, |log_2_FC| > 1, *P* < 0.05) within 3 kb of each gene’s promoter. Genes upregulated in melanoma are to the right, and genes with a more open chromatin region near the promoter in melanoma are on the top half. (B, C) All genes from ZFIN.org associated with NC (long list, Supplementary Table S3) with expression plotted with all accessible peaks (B) within 3 kb of the TSS (Pearson correlation coefficient *R* = 0.3951) and (C) only at the TSS (*R* = 0.6067). Points representing genes significantly differentially expressed (|log_2_FC| > 1, *P* < 0.05) in RNA-seq are in green, points representing peaks significantly differentially accessible in ATAC-seq (|log_2_FC| > 1, *P* < 0.05) are in black, points neither significantly expressed nor significantly accessible in blue, and points representing genes and peaks which are both significantly differentially expressed and significantly differentially accessible are in pink. (D, E) Genes associated with specific NC gene regulatory network stages discussed in the literature (shortlist, Supplementary Table S3) with expression plotted with all peaks (D) within 3 kb of the TSS (*R* = 0.6686) and (E) at the TSS with each point labeled with the gene name (*R* = 0.8488). Significance color scheme same as in (B, C).

To examine potential relationships between open chromatin and gene expression, we assessed the relationship between log_2_FC for differentially expressed genes and differentially accessible promoter peaks. As expected, the majority of genes with greater expression in melanoma had more accessibility with peaks within 3 kb of the promoter (*n* = 2386 pairs of genes and peaks; one gene can be linked to multiple ATAC-seq peaks), while genes with less expression were less accessible at the promoter (*n* = 499). Interestingly, 1213 genes had greater accessibility at the promoter in melanoma, yet had lower expression. We then focused on evaluating the relationship between accessibility and expression in NC genes using a “long list” of 329 genes associated with NC from ZFIN.org and a curated shortlist of 44 genes associated with specific NC developmental stages (Supplementary Tables S3 and S5). Utilizing the long list, we found a modest correlation (Pearson’s correlation coefficient *R* = 0.3951) between gene expression accessibility at promoter-proximal peaks within 3 kb of the promoter (Figure 4B). This relationship was strengthened when focusing only on peaks at the TSS (Figure 4C;*R* = 0.6067). Further, focusing specifically on genes previously shown to be involved in NC development, this association was even more pronounced (Figure 4D; *R* = 0.6686), with peaks at the promoter showing a clear correlation with expression (Figure 4E;*R* = 0.8488).

## Discussion

Here, we present a zebrafish model allowing for efficient isolation of melanocytes and melanoma cells and provide a resource capturing transcriptional and chromatin accessibility changes occurring *in vivo* during *de novo* tumor development. Independent replicates from genetically related animals add robustness to the dataset while limiting the background genetic complexity of a fully outbred population. This combined RNA- and ATAC-seq analysis of normal, premalignant, and transformed melanocytes/melanoma offers a tool for gene and pathway discovery in melanoma biology. Moreover, the tumor diversity with pigmentation status and tumor location (Supplementary Table S2) provides a comprehensive picture of cutaneous melanoma in a BRAF-driven, MITF-high model (Travnickova *et al.* 2019). Based on widely used QC metrics, we conclude that our RNA- and ATAC-seq data accurately reflect the *in vivo* transcriptional and chromatin accessibility state of the average premalignant BRAF/p53 mutant melanocyte and melanoma cell and provide an important and broadly useful tool for further investigating transcriptional and epigenetic programs underlying these related but crucially different pre- and fully malignant states. Similar studies have shed light on the epigenetic regulation of dysregulated genes that promote melanoma, such as *sox10* (Cunningham *et al.* 2021), or have been used to delineate developmental programs underlying erythroid differentiation (Ludwig *et al.* 2019). Studies have found a reactivation of an NC program in melanoma (Kaufman *et al.* 2016), and indeed we observed upregulation of NC genes such as *crestin*, *sox10*, *tfap2a*, *dlx2a*, among others. However, when we expanded the analysis to include NC genes segregated by stage of expression in NC, there was not a clear program upregulated in melanoma (*i.e.*, not all migratory NC markers were upregulated). This further supports a widespread dysregulation of expression programs in the disease state and begs the question of which consistent gene program is reactivated in melanoma. Nevertheless, there is an apparent relationship between accessibility at the TSS and gene expression of NC-associated genes (Figure 4E).

Interestingly, our dataset shows that in melanoma there are more genes with significant downregulation in melanoma relative to melanocytes, despite the presence of more accessible regions in the melanoma genome, supporting previous findings that points of control other than promoter accessibility, such as TF and chromatin regulatory protein abundances and activities, may be primary influencers of gene expression and thus changes in cell identity in the transition to a malignant state (Travnickova *et al.* 2019; Santoriello *et al.* 2020; Baggiolini *et al.* 2021; Fazio *et al.* 2021; Terranova *et al.* 2021). Indeed, it has been shown that very distal enhancers can control gene expression, indicating that open regions of chromatin may not necessarily regulate the most proximal gene (Lettice *et al.* 2003; Amano *et al.* 2009; Lacomme *et al.* 2018). Most changes in accessibility occur in distal nonpromoter regions (Supplementary Figure S4C; Thurman *et al.* 2012; Pliner *et al.* 2018; Friman *et al.* 2019), and deciphering the relationship between gene expression and *cis*-regulatory regions has been a topic of immense focus (Ackermann *et al.* 2016; Gonen *et al.* 2018; Zhao *et al.* 2019; Cai *et al.* 2020; Cunningham *et al.* 2021; Panigrahi and O’Malley 2021). Our study serves as a foundation to probe open chromatin regions and potential regulatory functions.

Furthermore, though all samples had an active melanocyte-specific program, genes typically associated with melanocytes were upregulated and more accessible in melanoma, consistent with a widespread dysregulation in additional gene programs in the melanoma cell state. We anticipate these data will contribute to ongoing functional analyses testing candidates that control the crucial epigenetic and transcriptional differences driving the transition between the normal and diseased cell state. 

## Data availability

Strains are available upon request. BigWig files can be found on the Gene Expression Ombnibus, accession no. GSE178803. Supplementary files submitted through figshare (https://doi.org/10.25387/g3.16841419).

## References

[jkab379-B1] Ackermann AM , Wang Z, Schug J, Naji A, Kaestner KH. 2016. Integration of ATAC-seq and RNA-seq identifies human alpha cell and beta cell signature genes. Mol Metab. 5:233–244. 26977395 10.1016/j.molmet.2016.01.002 PMC4770267

[jkab379-B2] Amano T , Sagai T, Tanabe H, Mizushina Y, Nakazawa H, et al 2009. Chromosomal dynamics at the Shh locus: limb bud-specific differential regulation of competence and active transcription. Dev Cell. 16:47–57. 19097946 10.1016/j.devcel.2008.11.011

[jkab379-B3] Anders S , Huber W. 2010. Differential expression analysis for sequence count data. Genome Biol. 11:R106. 20979621 10.1186/gb-2010-11-10-r106 PMC3218662

[jkab379-B4] Andrews S. 2010. FASTQC: a quality control tool for high throughput sequence data. http://www.Bioinformatics.Babraham.Ac.Uk/projects/fastqc (Accessed: 2021 November 10).

[jkab379-B5] Badal B , Solovyov A, Di Cecilia S, Chan JM, Chang L-W, et al 2017. Transcriptional dissection of melanoma identifies a high-risk subtype underlying TP53 family genes and epigenome deregulation. JCI Insight. 2:e92102. 10.1172/jci.insight.92102 PMC5414564 28469092

[jkab379-B6] Baggiolini A , Callahan S. J., Montal E, Weiss J. M., Trieu T, et al 2021. Developmental chromatin programs determine oncogenic competence in melanoma. Science. 373:eabc1048. 34516843 10.1126/science.abc1048 PMC9440978

[jkab379-B7] Barnett DW , Garrison EK, Quinlan AR, Stromberg MP, Marth GT. 2011. Bamtools. Bioinformatics. 27:1691–1692. 21493652 10.1093/bioinformatics/btr174 PMC3106182

[jkab379-B8] Baron M , Tagore M, Hunter MV, Kim IS, Moncada R, et al 2020. The stress-like cancer cell state is a consistent component of tumorigenesis. Cell Syst. 11:536–546.e7. 32910905 10.1016/j.cels.2020.08.018 PMC8027961

[jkab379-B9] Belote RL , Le D, Maynard A, Lang UE, Sinclair A, et al 2021. Human melanocyte development and melanoma dedifferentiation at single-cell resolution. Nat Cell Biol. 23:1035–1047. 34475532 10.1038/s41556-021-00740-8

[jkab379-B10] Buenrostro JD , Giresi PG, Zaba LC, Chang HY, Greenleaf WJ. 2013. Transposition of native chromatin for fast and sensitive epigenomic profiling of open chromatin, DNA-binding proteins and nucleosome position. Nat Methods. 10:1213–1218. 24097267 10.1038/nmeth.2688 PMC3959825

[jkab379-B11] Cai W , Huang J, Zhu Q, Li BE, Seruggia D, et al 2020. Enhancer dependence of cell-type-specific gene expression increases with developmental age. Proc Natl Acad Sci U S A. 117:21450–21458. 32817427 10.1073/pnas.2008672117 PMC7474592

[jkab379-B12] Cancer Genome Atlas Network. 2015. Genomic classification of cutaneous melanoma. Cell. 161:1681–1696. 26091043 10.1016/j.cell.2015.05.044 PMC4580370

[jkab379-B13] Carroll TS , Liang Z, Salama R, Stark R, de Santiago I. 2014. Impact of artifact removal on ChIP quality metrics in ChIP-seq and ChIP-exo data. Front Genet. 5:75–75. 24782889 10.3389/fgene.2014.00075 PMC3989762

[jkab379-B14] Ceol CJ , Houvras Y, Jane-Valbuena J, Bilodeau S, Orlando DA, et al 2011. The histone methyltransferase SETDB1 is recurrently amplified in melanoma and accelerates its onset. Nature. 471:513–517. 21430779 10.1038/nature09806 PMC3348545

[jkab379-B15] Cirenajwis H , Ekedahl H, Lauss M, Harbst K, Carneiro A, et al 2015. Molecular stratification of metastatic melanoma using gene expression profiling: prediction of survival outcome and benefit from molecular targeted therapy. Oncotarget. 6:12297–12309. 25909218 10.18632/oncotarget.3655 PMC4494939

[jkab379-B16] Cisarova K , Folcher M, El Zaoui I, Pescini-Gobert R, Peter VG, et al 2020. Genomic and transcriptomic landscape of conjunctival melanoma. PLoS Genet. 16:e1009201. 33383577 10.1371/journal.pgen.1009201 PMC7775126

[jkab379-B17] Corces MR , Buenrostro JD, Wu B, Greenside PG, Chan SM, et al 2016. Lineage-specific and single-cell chromatin accessibility charts human hematopoiesis and leukemia evolution. Nat Genet. 48:1193–1203. 27526324 10.1038/ng.3646 PMC5042844

[jkab379-B18] Cronin JC , Watkins-Chow DE, Incao A, Hasskamp JH, Schönewolf N, et al 2013. SOX10 ablation arrests cell cycle, induces senescence, and suppresses melanomagenesis. Cancer Res. 73:5709–5718. 23913827 10.1158/0008-5472.CAN-12-4620 PMC3803156

[jkab379-B19] Cunningham RL , Kramer ET, DeGeorgia SK, Godoy PM, Zarov AP, et al 2021. Functional *in vivo* characterization of *sox10* enhancers in neural crest and melanoma development. Commun Biol. 4:695. 34099848 10.1038/s42003-021-02211-0 PMC8184803

[jkab379-B20] Dobin A , Davis CA, Schlesinger F, Drenkow J, Zaleski C, et al 2013. Star: ultrafast universal RNA-seq aligner. Bioinformatics. 29:15–21. 23104886 10.1093/bioinformatics/bts635 PMC3530905

[jkab379-B21] Durante MA , Rodriguez DA, Kurtenbach S, Kuznetsov JN, Sanchez MI, et al 2020. Single-cell analysis reveals new evolutionary complexity in uveal melanoma. Nat Commun. 11:496. 31980621 10.1038/s41467-019-14256-1 PMC6981133

[jkab379-B22] Ennen M , Keime C, Kobi D, Mengus G, Lipsker D, et al 2015. Single-cell gene expression signatures reveal melanoma cell heterogeneity. Oncogene. 34:3251–3263. 25132268 10.1038/onc.2014.262

[jkab379-B23] Fazio M , van Rooijen E, Dang M, van de Hoek G, Ablain J, et al 2021. SATB2 induction of a neural crest mesenchyme-like program drives melanoma invasion and drug resistance. Elife. 10:e64370. 33527896 10.7554/eLife.64370 PMC7880683

[jkab379-B24] Friman ET , Deluz C, Meireles-Filho ACA, Govindan S, Gardeux V, et al 2019. Dynamic regulation of chromatin accessibility by pluripotency transcription factors across the cell cycle. Elife. 8:e50087. 31794382 10.7554/eLife.50087 PMC6890464

[jkab379-B25] Gan Y , Li N, Zou G, Xin Y, Guan J. 2018. Identification of cancer subtypes from single-cell RNA-seq data using a consensus clustering method. BMC Med Genomics. 11:117. 30598115 10.1186/s12920-018-0433-z PMC6311928

[jkab379-B26] Garg M , Couturier D-L, Nsengimana J, Fonseca NA, Wongchenko M, et al 2021. Tumour gene expression signature in primary melanoma predicts long-term outcomes. Nat Commun. 12:1137. 33602918 10.1038/s41467-021-21207-2 PMC7893180

[jkab379-B27] Gonen N , Futtner CR, Wood S, Garcia-Moreno SA, Salamone IM, et al 2018. Sex reversal following deletion of a single distal enhancer of *Sox9*. Science. 360:1469–1473. 29903884 10.1126/science.aas9408 PMC6034650

[jkab379-B28] Haltaufderhyde KD , Oancea E. 2014. Genome-wide transcriptome analysis of human epidermal melanocytes. Genomics. 104:482–489. 25451175 10.1016/j.ygeno.2014.09.010 PMC4594949

[jkab379-B29] Harbst K , Staaf J, Lauss M, Karlsson A, Måsbäck A, et al 2012. Molecular profiling reveals low- and high-grade forms of primary melanoma. Clin Cancer Res. 18:4026–4036. 22675174 10.1158/1078-0432.CCR-12-0343 PMC3467105

[jkab379-B30] Hayward NK , Wilmott JS, Waddell N, Johansson PA, Field MA, et al 2017. Whole-genome landscapes of major melanoma subtypes. Nature. 545:175–180. 28467829 10.1038/nature22071

[jkab379-B31] Heinz S , Benner C, Spann N, Bertolino E, Lin YC, et al 2010. Simple combinations of lineage-determining transcription factors prime cis-regulatory elements required for macrophage and B cell identities. Mol Cell. 38:576–589. 20513432 10.1016/j.molcel.2010.05.004 PMC2898526

[jkab379-B32] Hodis E , Watson IR, Kryukov GV, Arold ST, Imielinski M, et al 2012. A landscape of driver mutations in melanoma. Cell. 150:251–263. 22817889 10.1016/j.cell.2012.06.024 PMC3600117

[jkab379-B33] Jönsson G , Busch C, Knappskog S, Geisler J, Miletic H, et al 2010. Gene expression profiling-based identification of molecular subtypes in stage IV melanomas with different clinical outcome. Clin Cancer Res. 16:3356–3367. 20460471 10.1158/1078-0432.CCR-09-2509

[jkab379-B34] Kaufman CK , Mosimann C, Fan ZP, Yang S, Thomas AJ, et al 2016. A zebrafish melanoma model reveals emergence of neural crest identity during melanoma initiation. Science. 351:aad2197. 26823433 10.1126/science.aad2197 PMC4868069

[jkab379-B35] Kent WJ , Zweig AS, Barber G, Hinrichs AS, Karolchik D. 2010. Bigwig and bigbed: enabling browsing of large distributed datasets. Bioinformatics. 26:2204–2207. 20639541 10.1093/bioinformatics/btq351 PMC2922891

[jkab379-B36] Kim S , Yu N-K, Kaang B-K. 2015. CTCF as a multifunctional protein in genome regulation and gene expression. Exp Mol Med. 47:e166. 26045254 10.1038/emm.2015.33 PMC4491725

[jkab379-B37] Kolde R. 2015. Pheatmap: pretty heatmaps [software]. https://CRAN.R-project.org/package=pheatmap (Accessed: 2021 November 10).

[jkab379-B38] Kunz M , Löffler-Wirth H, Dannemann M, Willscher E, Doose G, et al 2018. RNA-seq analysis identifies different transcriptomic types and developmental trajectories of primary melanomas. Oncogene. 37:6136–6151. 29995873 10.1038/s41388-018-0385-y

[jkab379-B39] Lacomme M , Medevielle F, Bourbon H-M, Thierion E, Kleinjan D-J, et al 2018. A long range distal enhancer controls temporal fine-tuning of PAX6 expression in neuronal precursors. Dev Biol. 436:94–107. 29486153 10.1016/j.ydbio.2018.02.015

[jkab379-B40] Lauss M , Nsengimana J, Staaf J, Newton-Bishop J, Jönsson G. 2016. Consensus of melanoma gene expression subtypes converges on biological entities. J Invest Dermatol. 136:2502–2505. 27345472 10.1016/j.jid.2016.05.119

[jkab379-B41] Lettice LA , Heaney SJ, Purdie LA, Li L, de Beer P, et al 2003. A long-range Shh enhancer regulates expression in the developing limb and fin and is associated with preaxial polydactyly. Hum Mol Genet. 12:1725–1735. 12837695 10.1093/hmg/ddg180

[jkab379-B42] Li B , Dewey CN. 2011. RSEM: accurate transcript quantification from RNA-seq data with or without a reference genome. BMC Bioinformatics. 12:323. 21816040 10.1186/1471-2105-12-323 PMC3163565

[jkab379-B43] Li H , Durbin R. 2009. Fast and accurate short read alignment with burrows-wheeler transform. Bioinformatics. 25:1754–1760. 19451168 10.1093/bioinformatics/btp324 PMC2705234

[jkab379-B44] Li H , Handsaker B, Wysoker A, Fennell T, Ruan J, et al; 1000 Genome Project Data Processing Subgroup. 2009. The sequence alignment/map format and samtools. Bioinformatics. 25:2078–2079. 19505943 10.1093/bioinformatics/btp352 PMC2723002

[jkab379-B45] Li Q , Brown JB, Huang H, Bickel PJ. 2011. Measuring reproducibility of high-throughputexperiments. Ann Appl Stat. 5:1752–1779.

[jkab379-B46] Li X , Karras P, Torres R, Rambow F, van den Oord J, et al 2020. Disseminated melanoma cells transdifferentiate into endothelial cells in intravascular niches at metastatic sites. Cell Rep. 31:107765. 32553158 10.1016/j.celrep.2020.107765

[jkab379-B47] Ludwig LS , Lareau CA, Bao EL, Nandakumar SK, Muus C, et al 2019. Transcriptional states and chromatin accessibility underlying human erythropoiesis. Cell Rep. 27:3228–3240.e7. 31189107 10.1016/j.celrep.2019.05.046 PMC6579117

[jkab379-B48] Marie KL , Sassano A, Yang HH, Michalowski AM, Michael HT, et al 2020. Melanoblast transcriptome analysis reveals pathways promoting melanoma metastasis. Nat Commun. 11:333. 31949145 10.1038/s41467-019-14085-2 PMC6965108

[jkab379-B49] Martin M. 2011. Cutadapt removes adapter sequences from high-throughput sequencing reads. EMBnet J. 17:10–12.

[jkab379-B50] McKenna A , Hanna M, Banks E, Sivachenko A, Cibulskis K, et al 2010. The genome analysis toolkit: a mapreduce framework for analyzing next-generation DNA sequencing data. Genome Res. 20:1297–1303. 20644199 10.1101/gr.107524.110 PMC2928508

[jkab379-B51] Mohamed A , Gonzalez RS, Lawson D, Wang J, Cohen C. 2013. SOX10 expression in malignant melanoma, carcinoma, and normal tissues. Appl Immunohistochem Mol Morphol. 21:506–510. 23197006 10.1097/PAI.0b013e318279bc0a

[jkab379-B52] Mootha VK , Lindgren CM, Eriksson K-F, Subramanian A, Sihag S, et al 2003. PGC-1α-responsive genes involved in oxidative phosphorylation are coordinately downregulated in human diabetes. Nat Genet. 34:267–273. 12808457 10.1038/ng1180

[jkab379-B53] Nsengimana J , Laye J, Filia A, Walker C, Jewell R, et al 2015. Independent replication of a melanoma subtype gene signature and evaluation of its prognostic value and biological correlates in a population cohort. Oncotarget. 6:11683–11693. 25871393 10.18632/oncotarget.3549 PMC4484486

[jkab379-B54] Panigrahi A , O’Malley BW. 2021. Mechanisms of enhancer action: the known and the unknown. Genome Biol. 22:108. 33858480 10.1186/s13059-021-02322-1 PMC8051032

[jkab379-B55] Patton EE , Widlund HR, Kutok JL, Kopani KR, Amatruda JF, et al 2005. BRAF mutations are sufficient to promote nevi formation and cooperate with p53 in the genesis of melanoma. Curr Biol. 15:249–254. 15694309 10.1016/j.cub.2005.01.031

[jkab379-B56] Pliner HA , Packer JS, McFaline-Figueroa JL, Cusanovich DA, Daza RM, et al 2018. Cicero predicts cis-regulatory DNA interactions from single-cell chromatin accessibility data. Mol Cell. 71:858–871.e8. 30078726 10.1016/j.molcel.2018.06.044 PMC6582963

[jkab379-B57] Quinlan AR , Hall IM. 2010. Bedtools: a flexible suite of utilities for comparing genomic features. Bioinformatics. 26:841–842. 20110278 10.1093/bioinformatics/btq033 PMC2832824

[jkab379-B58] Rabbie R , Ferguson P, Molina-Aguilar C, Adams DJ, Robles-Espinoza CD. 2019. Melanoma subtypes: genomic profiles, prognostic molecular markers and therapeutic possibilities. J Pathol. 247:539–551. 30511391 10.1002/path.5213 PMC6492003

[jkab379-B59] Rambow F , Rogiers A, Marin-Bejar O, Aibar S, Femel J, et al 2018. Toward minimal residual disease-directed therapy in melanoma. Cell. 174:843–855.e19. 30017245 10.1016/j.cell.2018.06.025

[jkab379-B60] Reemann P , Reimann E, Ilmjärv S, Porosaar O, Silm H, et al 2014. Melanocytes in the skin–comparative whole transcriptome analysis of main skin cell types. PLoS One. 9:e115717. 25545474 10.1371/journal.pone.0115717 PMC4278762

[jkab379-B61] Reuben A , Spencer CN, Prieto PA, Gopalakrishnan V, Reddy SM, et al 2017. Genomic and immune heterogeneity are associated with differential responses to therapy in melanoma. NPJ Genomic Med. 2:10. 10.1038/s41525-017-0013-8 PMC5557036 28819565

[jkab379-B62] Sakabe NJ , Aneas I, Knoblauch N, Sobreira DR, Clark N, et al 2020. Transcriptome and regulatory maps of decidua-derived stromal cells inform gene discovery in preterm birth. Sci Adv. 6:eabc8696. 33268355 10.1126/sciadv.abc8696 PMC7710387

[jkab379-B63] Santoriello C , Sporrij A, Yang S, Flynn RA, Henriques T, et al 2020. RNA helicase ddx21 mediates nucleotide stress responses in neural crest and melanoma cells. Nat Cell Biol. 22:372–379. 32231306 10.1038/s41556-020-0493-0 PMC7185069

[jkab379-B64] Shakhova O , Zingg D, Schaefer SM, Hari L, Civenni G, et al 2012. Sox10 promotes the formation and maintenance of giant congenital naevi and melanoma. Nat Cell Biol. 14:882–890. 22772081 10.1038/ncb2535

[jkab379-B65] Simões-Costa M , Bronner ME. 2015. Establishing neural crest identity: a gene regulatory recipe. Development. 142:242–257. 25564621 10.1242/dev.105445 PMC4302844

[jkab379-B66] Stark R , Brown G. 2011. DiffBind: differential binding analysis of ChIP-seq peak data. https://bioconductor.org/packages/release/bioc/html/DiffBind.html. (Accessed: 2021 November 10).

[jkab379-B67] Subramanian A , Tamayo P, Mootha VK, Mukherjee S, Ebert BL, et al 2005. Gene set enrichment analysis: a knowledge-based approach for interpreting genome-wide expression profiles. Proc Natl Acad Sci USA. 102:15545–15550. 16199517 10.1073/pnas.0506580102 PMC1239896

[jkab379-B68] Terranova CJ , Tang M, Maitituoheti M, Raman AT, Ghosh AK, et al 2021. Reprogramming of bivalent chromatin states in NRAS mutant melanoma suggests PRC2 inhibition as a therapeutic strategy. Cell Rep. 36:109410. 34289358 10.1016/j.celrep.2021.109410 PMC8369408

[jkab379-B69] Thurman RE , Rynes E, Humbert R, Vierstra J, Maurano MT, et al 2012. The accessible chromatin landscape of the human genome. Nature. 489:75–82. 22955617 10.1038/nature11232 PMC3721348

[jkab379-B70] Tirosh I , Venteicher AS, Hebert C, Escalante LE, Patel AP, et al 2016. Single-cell RNA-seq supports a developmental hierarchy in human oligodendroglioma. Nature. 539:309–313. 27806376 10.1038/nature20123 PMC5465819

[jkab379-B71] Travnickova J , Patton EE. 2021. Deciphering melanoma cell states and plasticity with zebrafish models. J Invest Dermatol. 141:1389–1394. 33340501 10.1016/j.jid.2020.12.007 PMC8168147

[jkab379-B72] Travnickova J , Wojciechowska S, Khamseh A, Gautier P, Brown DV, et al 2019. Zebrafish MITF-low melanoma subtype models reveal transcriptional subclusters and MITF-independent residual disease. Cancer Res. 79:5769–5784. 31582381 10.1158/0008-5472.CAN-19-0037 PMC7116150

[jkab379-B73] Tsoi J , Robert L, Paraiso K, Galvan C, Sheu KM, et al 2018. Multi-stage differentiation defines melanoma subtypes with differential vulnerability to drug-induced iron-dependent oxidative stress. Cancer Cell. 33:890–904.e5. 29657129 10.1016/j.ccell.2018.03.017 PMC5953834

[jkab379-B74] Venkatesan AM , Vyas R, Gramann AK, Dresser K, Gujja S, et al 2018. Ligand-activated BMP signaling inhibits cell differentiation and death to promote melanoma. J Clin Invest. 128:294–308. 29202482 10.1172/JCI92513 PMC5749509

[jkab379-B75] White RM , Cech J, Ratanasirintrawoot S, Lin CY, Rahl PB, et al 2011. DHODH modulates transcriptional elongation in the neural crest and melanoma. Nature. 471:518–522. 21430780 10.1038/nature09882 PMC3759979

[jkab379-B76] Wickham H. 2016. Ggplot2: Elegant Graphics for Data Analysis. New York, NY: Springer-Verlag.

[jkab379-B77] Williams RM , Candido-Ferreira I, Repapi E, Gavriouchkina D, Senanayake U, et al 2019. Reconstruction of the global neural crest gene regulatory network in vivo. Dev Cell. 51:255–276.e7. 31639368 10.1016/j.devcel.2019.10.003 PMC6838682

[jkab379-B78] Wouters J , Kalender-Atak Z, Minnoye L, Spanier KI, De Waegeneer M, et al 2020. Robust gene expression programs underlie recurrent cell states and phenotype switching in melanoma. Nat Cell Biol. 22:986–998. 32753671 10.1038/s41556-020-0547-3

[jkab379-B79] Yen J , White RM, Wedge DC, Van Loo P, de Ridder J, et al 2013. The genetic heterogeneity and mutational burden of engineered melanomas in zebrafish models. Genome Biol. 14: R113. 24148783 10.1186/gb-2013-14-10-r113 PMC3983654

[jkab379-B80] Zhang Y , Liu T, Meyer CA, Eeckhoute J, Johnson DS, et al 2008. Model-based analysis of ChIP-seq (macs). Genome Biol. 9:R137. 18798982 10.1186/gb-2008-9-9-r137 PMC2592715

[jkab379-B81] Zhao Y , Zheng D, Cvekl A. 2019. Profiling of chromatin accessibility and identification of general cis-regulatory mechanisms that control two ocular lens differentiation pathways. Epigenetics Chromatin. 12:27. 31053165 10.1186/s13072-019-0272-y PMC6498704

